# Evaluation of Safety-Related Outcomes of One-Segment and More-Than-One-Segment High-Level Hepatectomy in Hepatocellular Carcinoma Based on the Japanese Board Certification System

**DOI:** 10.1007/s00268-022-06467-3

**Published:** 2022-02-12

**Authors:** Akihisa Hojo, Hisashi Nakayama, Yukiyasu Okamura, Tokio Higaki, Masamichi Moriguchi, Osamu Aramaki, Shitaro Yamazaki, Tadatoshi Takayama

**Affiliations:** Division of Digestive Surgery, Department of Surgery, Nihon University School of Medicine, 30-1 Oyaguchikami-cho, Itabashi-ku, Tokyo 173-8610 Japan

## Abstract

**Background:**

We evaluated the impact of the Japanese board certification system for expert surgeons (JBCSES) on complications and survival outcomes in hepatectomy for hepatocellular carcinoma.

**Methods:**

The postoperative outcomes of 493 patients who underwent high-level liver surgery involving one-segment (OSeg) hepatectomy and more-than-one-segment (MOSeg) resection were compared before and after JBCSES establishment. After the establishment of the JBCSES, the patients’ postoperative outcomes were compared using propensity score matching (PSM) to determine the influence of expert surgeons.

**Results:**

The establishment of the JBCSES was associated with a decrease in the overall postoperative complication rates after high-level liver surgery from 50.2 to 38.1% (*P* = 0.008) and a decrease in Clavien–Dindo class ≥ IIIb complications from 10.2 to 5.0% (*P* = 0.035). The 90-day mortality rate decreased from 5.1 to 0.7% (*P* = 0.003), and the 5-year survival rate increased from 51.4 to 63.9% (*P* = 0.009). Using PSM, a comparison of OSeg hepatectomies that involved expert surgeons (*n* = 48) and those that did not (*n* = 48) showed significantly lower intraoperative blood loss in surgeries involving an expert surgeon (mean, 340 vs. 473 mL; *P* = 0.033). There were no significant differences in complication rates or long-term prognosis between these groups. A comparison of MOSeg hepatectomies that involved expert surgeons (*n* = 26) and those that did not (*n* = 26) showed no significant difference in surgical factors, complications, or overall survival between the two groups.

**Conclusions:**

After establishment of the JBCSES, postoperative complication rates and mortality rates decreased and survival rates increased following liver surgery. Expert surgeon participation significantly decreased intraoperative blood loss during OSeg hepatectomies.

## Introduction

The Japanese Society of Hepato-Biliary-Pancreatic Surgery (JSHBPS) approved and established a Japanese board certification system for expert surgeons (JBCSES) in 2008 to increase the safety and reliability of high-level hepato-biliary-pancreatic surgeries [1]. Liver resections are considered very difficult, and a study on hepatectomy of more than one segment (MOSeg) using data from the National Clinical Database (NCD) of Japan revealed that participation of an expert surgeon resulted in lower surgical mortality than non-participation (3.5% vs. 4.3%, *P* = 0.012) [2]. Surgical time was also significantly longer, but blood loss volumes were lower for MOSeg hepatectomies in diseases such as hepatocellular carcinoma (HCC), liver metastasis, intrahepatic cholangiocarcinoma, hilar cholangiocarcinoma, and gallbladder cancer that involved expert surgeons than for those that did not. The post-hepatectomy mortality rate in high-volume hospitals is lower than that in low-volume hospitals in the United States [3], and this concept has been applied by the JBCSES to certify hospitals performing many high-level surgeries.

Hepatectomy is standard therapy in HCC; however, many patients experience chronic hepatitis and cirrhosis [4]. Therefore, major resections such as sectionectomy and hemihepatectomy (MOSeg hepatectomies) [5, 6] are better avoided to preserve liver function in patients with HCC with severe cirrhosis, who are at higher risk of postoperative liver failure [7]. Therefore, anatomical segmentectomy was developed as an oncological radical resection method that can be performed without major impairment of postoperative liver function. First, Couinaud proposed the liver’s theoretical segmental anatomy from an embryological perspective [8]. Then, Makuuchi et al. performed one-segment (OSeg) hepatectomy by staining the portal branch [9]. Both OSeg and MOSeg hepatectomies are methods included in the JBCSES, but the two involve completely different procedures [10]. In MOSeg hepatectomy, the hepatic hilar transection method [11] and the Glissonean pedicle transection method [12, 13] are used, but in OSeg hepatectomy, the staining method is common [9, 14]. However, data comparing the safety and efficacy of OSeg and MOSeg hepatectomies to assess the JBCSES are insufficient.

The first purpose was to evaluate the trends of changes in complication frequencies and patient survival before and after the establishment of the JBCSES. Second, we aimed to evaluate the impact of expert surgeons using propensity score matching for OSeg and MOSeg hepatectomies. We assessed operative outcomes based on whether expert surgeons participated in the surgery. The volumes of intraoperative bleeding and blood transfusions, length of hospital stay, and frequency of postoperative complications were treated as short-term outcomes, and the cumulative survival rate was treated as a long-term outcome.

## Material and methods

### Patients and high-level liver surgery criteria

**Fig. 1:**
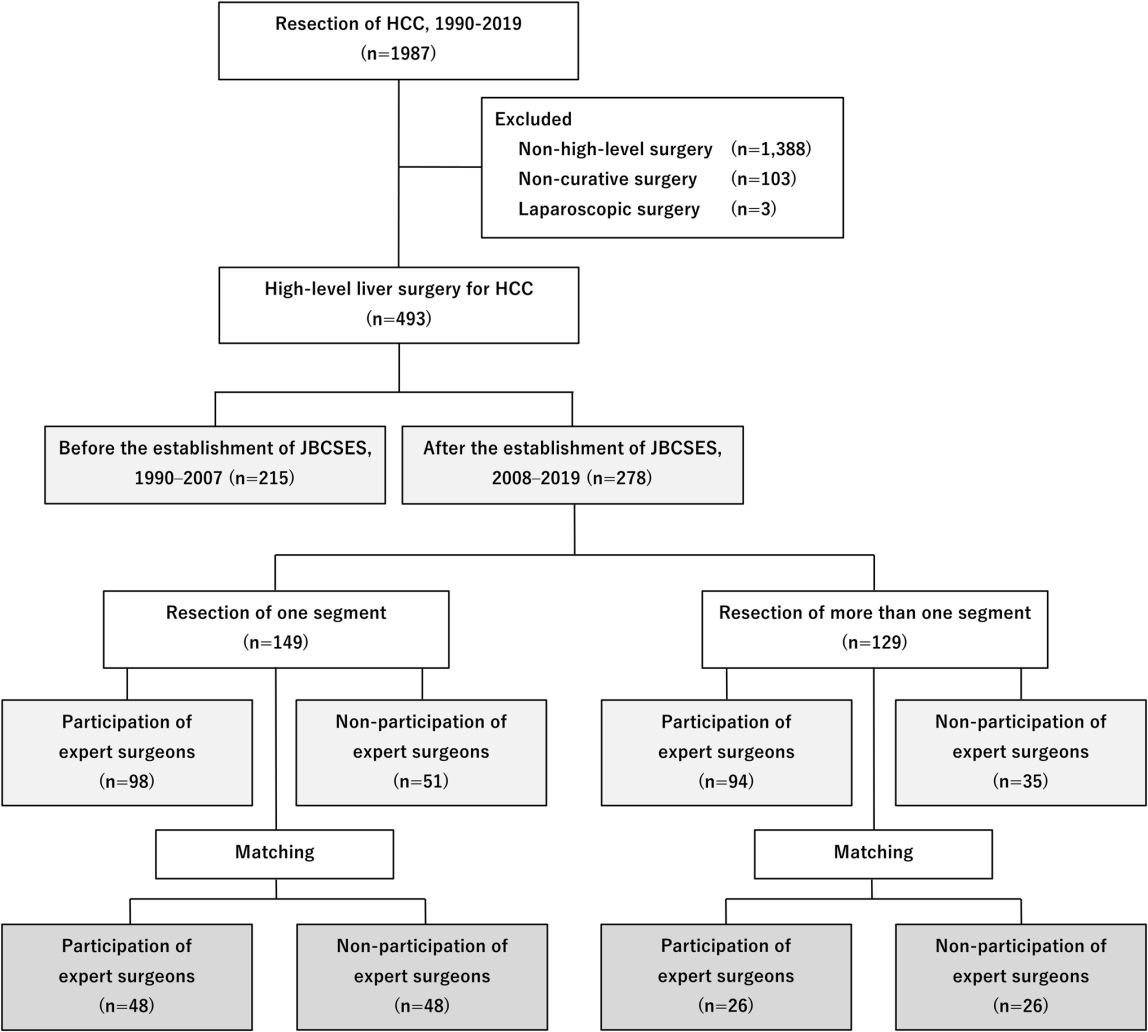
Flow diagram depicting patient recruitment. HCC, hepatocellular carcinoma; JBCSES, Japanese board certification system for expert surgeons

Of the 1,987 patients with HCC who underwent hepatectomies at Nihon University Itabashi Hospital from 1990 to 2019, 493 who underwent high-level liver surgery were included (Fig. 1). Hepatic trisectionectomy, hemihepatectomy [13, 15, 16], central bisectionectomy of the liver [17], hepatic sectionectomy (except for lateral sectionectomy) [18], and hepatic segmentectomy (S1, S2, S3, S5, S6, S7, and S8) procedures were considered high-level liver surgeries, as defined by the JSHBPS [1]. Patients who underwent laparoscopic hepatectomy were excluded. Patient who underwent non-curative hepatectomy procedures, corresponding to curability *C* of the General Rules for the Clinical and Pathological Study of Primary Liver Cancer [19, 20], were also excluded.

Patients were classified according to when they underwent surgery: 1990–2007, which was prior to the approval of the JBCSES (first period; *n* = 215), and 2008–2019, after its approval (second period; *n* = 278). Hepatic segmentectomy was defined as OSeg hepatectomy, whereas hepatectomy of more than one segment was defined as MOSeg hepatectomy. Three expert surgeons (including board-certified instructor) certified by the JBCSES in 2008, two certified from 2009 to 2013, and four certified from 2014 to 2019 performed the hepatectomies. Fifteen operators not certified as expert surgeons performed hepatectomies from 2008 to 2019. The study design conformed to the ethical guidelines of the Declaration of Helsinki and was approved by the institutional review board of Nihon University (ID: RK-200702-1). Written informed consent was obtained before enrollment in the study.

### Surgical procedure and postoperative follow-up

Surgical indication and procedures consisted of determining the extent of hepatectomy based on the indocyanine green retention rate at 15 min (ICG-R_15_) values [21], identification of liver segments by staining [9, 14], Pringle’s maneuver [22, 23], and Pean forceps fracture method for liver parenchyma transection [24]. Postoperative complications were classified using modified Clavien–Dindo grading [25]. Thirty-day mortality was defined as death within 30 days of hepatectomy, regardless of whether it occurred during hospitalization or after discharge. Surgical mortality included all patients who died within the index hospitalization period (up to 90 days) and within 30 days after discharge. All patients were followed up for postoperative recurrence. Alpha-fetoprotein and des-gamma-carboxy prothrombin tumor marker tests, abdominal ultrasound, and dynamic contrast-enhanced computed tomography were performed every 3 months.

### Propensity score matching and statistical analysis

Propensity matching was used to examine the effect of expert surgeon participation on the short- and long-term surgical outcomes among patients who underwent OSeg or MOSeg hepatectomies after 2008, when the JBCSES was implemented. We performed a one-to-one matching analysis comparing expert surgeon participation to non-participation on the basis of estimated propensity scores of each patient using a caliper width of 0.2 [26, 27]. Potential factors influencing surgical decisions include age (≥60 or <60 years), sex (male or female), HBsAg (positive or negative), HCVAb, platelet count (≥10^4/^μL or <10^4^/μL), plasma albumin levels (≥3.5 or <3.5 g/dL), total bilirubin levels (>0.7 or ≤0.7 mg/dL), prothrombin activity levels (≥70% or <70%), ICG-R_15_ levels (≥10% or <10%), tumor size (≥5 or <5 cm), and tumor count (single or multiple). These 11 variables were chosen to generate a propensity score using logistic regression modeling.

Clinical categorical and continuous variables were compared using Fisher’s exact and Wilcoxon’s rank-sum tests, respectively. Kaplan–Meier survival curves were created and compared using the log-rank test. Statistical significance was set at *P* < 0.05. Logistic regression analysis was used for the multivariate analysis of factors contributing to intraoperative bleeding. All statistical analyses and propensity score matching were performed using SPSS version 22 (IBM Corp., Armonk, NY, USA).

## Results

### Comparison of high-level liver surgery before and after JBCSES implementation

**Fig. 2:**
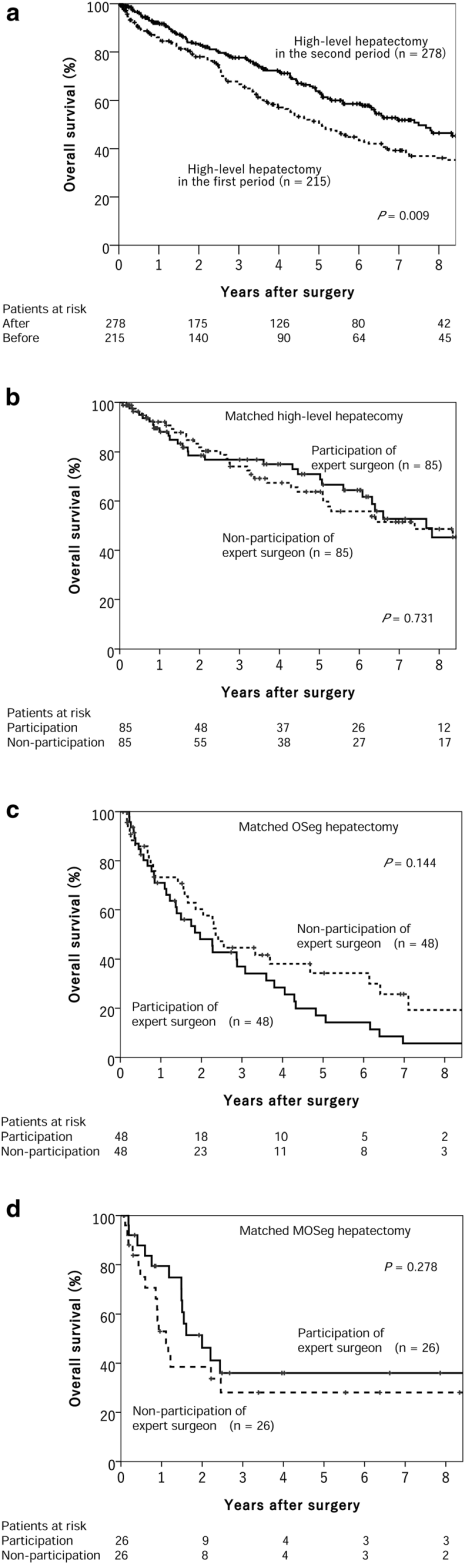
**a** Overall survival rate of patients who underwent high-level liver surgery for hepatocellular carcinoma before (first period) and after (second period) the establishment of the board certification system for expert surgeon. **b** Influence of expert surgeon for overall survival rate of matched patients who underwent high-level hepatectomy. **c** Influence of expert surgeon for overall survival rate of matched patients who underwent hepatectomy of one segment (OSeg). **d** Influence of expert surgeons for overall survival rate of matched patients who underwent hepatectomy of more than one segment (MOSeg)

Of the 1,987 patients who underwent hepatectomy for HCC between 1990 and 2019, 493 underwent high-level liver surgeries, 215 and 278 of which were performed in the first and second periods, respectively (Fig. 1). Patients treated in the second period were older than those in the first period but had lower rates of cirrhosis and esophageal varices (Table 1). Patients treated in the second period had higher platelet counts and albuminemia than those in the first period, but no significant difference was observed in ICG-R_15_ values or Child–Pugh grades. In the second period, surgical time and Pringle’s time were longer and blood loss and transfusion volumes were significantly lower than in the first period. The overall complication incidence and frequency of severe complications were significantly lower in the second period than in the first period. Surgical mortality decreased from 5.1 to 0.7% (*P* = 0.003). The median follow-up time for all 493 patients was 4.4 [range, 0.1–18.6] years; for the first and second periods, it was 3.4 [range, 0.1–18.6] and 3.5 [range, 0.1–12.2] years, respectively. The 5-year overall survival rate in the second period was significantly better at 63.9%, compared with 51.4% in the first period (*P* = 0.009) (Fig. 2a).

**Table 1:** Characteristics of patients who underwent Japanese board-certified high-level liver surgery

Characteristics	Entire study population (*n* = 493)	Board certification system		*P* value
		Before establishment (*n* = 215)	After establishment (*n* = 278)	
*Profiles*				
Age, years, mean	65.2	63.0	66.6	<0.001
Male sex, %	80.5	83.7	78.1	0.136
PS 0, %	99.0	98.6	99.3	0.657
BMI, mean	23.5	23.0	23.8	0.040
*Comorbidity*				
Alcohol habitually, %	30.6	37.7	25.2	0.003
Smoking, %	61.1	69.8	54.3	0.001
Liver cirrhosis, %	27.4	37.7	19.4	<0.001
Esophageal varices, %	16.6	24.7	10.4	<0.001
*Preoperative laboratory factors*				
HBsAg + , %	18.9	19.5	18.3	0.817
HCVAb + , %	48.7	61.9	38.5	<0.001
Hemoglobin, g/dL, mean	13.4	13.1	13.6	<0.001
Platelets, 10^4^/μL, mean	17.6	15.7	18.9	<0.001
Albumin, g/dL, mean	4.0	3.9	4.1	<0.001
Bilirubin, mg/dL, mean	0.67	0.70	0.69	0.631
AST, IU/L, mean	47.4	52.5	44.9	0.003
Prothrombin, %, mean	93.3	90.7	94.6	<0.001
ICG-R_15_, %, mean	13.0	13.2	13.0	0.183
Alpha fetoprotein, ng/mL, mean	4918	15,121	4962	0.001
DCP, mAU/mL, mean	4114	1755	5620	<0.001
Child–Pugh, A, %	97.6	96.7	98.2	0.380
*Surgical factors*				
OS/MOS hepatectomy, n	277/216	128/87	149/129	0.201
Surgical time, minutes, mean	366.6	319.7	393.0	<0.001
Pringle’s time, minutes, mean	81.8	64.0	90.3	<0.001
Blood loss, mL, mean	688.9	972.6	539.2	<0.001
Blood transfusion, mL, mean	85.4	114.5	67.3	0.001
*Tumor-related factors*				
Tumor number, single, %	76.3	75.3	77.0	0.671
Tumor size, cm, mean	5.3	5.0	5.5	0.890
Surgical margin, mm, mean	5.2	5.4	5.0	0.052
Complications, n (%)	214 (43.4%)	108 (50.2)	106 (38.1)	0.008
Clavien–Dindo grade				0.035
Grade I–IIIa, %	92.7	89.8	95.0	
Grade IIIb–V, %	7.3	10.2	5.0	
*Morbidity and mortality*				
30-day mortality, %	0.6	0.9	0.4	0.583
Surgical mortality, %	2.6	5.1	0.7	0.003
Hospital stays, day, mean	19.2	28.1	14.7	<0.001

*PS* performance status, *BMI* body mass index, *HBsAg* hepatitis B surface antigen, *HCVAb* hepatitis C virus antibody, *AST* Aspartate transaminase, *ICG-R15* indocyanine green retention rate at 15 min, *DCP* des-gamma-carboxyprothrombin, OS hepatectomy, hepatic segmentectomy so-called one-segment hepatectomy; MOS hepatectomy; hepatectomy of more than one segment

In the second period, expert surgeons participated in 192 high-level hepatectomies and did not participate in 86. Using propensity score matching, we compared 85 cases of expert surgeon participation and 85 cases of non-participation, and there was no significant difference for overall survival between the two groups (Fig. 2b).

### Impact of expert surgeons on OSeg hepatectomies

In the second period, expert surgeons participated in 98 OSeg hepatectomies and did not participate in 51. To evaluate their impact properly, propensity score matching was used to create 48 cases of expert surgeon participation and 48 cases of non-participation (Table 2). Patient profiles, comorbidities, and preoperative laboratory factors were evenly distributed after matching. The mean intraoperative bleeding volume was lower in procedures performed with expert surgeon participation than in those without (340 and 473 mL, respectively; *P* = 0.033). There were no significant differences in complication rates, length of stay, or long-term prognosis between the two groups (Fig. 2c).

**Table 2:** Impact of expert surgeon participation on OS hepatectomy before and after propensity score matching

Characteristic	Before matching			After matching		
	Participation (*n* = 98)	Non-participation (*n* = 51)	*P* value	Participation (*n* = 48)	Non-participation (*n* = 48)	*P* value
*Profiles*						
Age, years, mean	66.9	65.0	0.103	66.3	65.2	0.354
Male sex, %	76.5	74.5	0.841	68.8	72.9	0.823
PS 0, %	100	100	NS	100	100	NS
BMI, mean	23.7	24.0	0.533	23.2	23.8	0.278
*Comorbidity*						
Alcohol habitually, %	24.5	21.6	0.839	25.0	20.8	0.809
Smoking, %	54.1	37.3	0.059	52.1	37.5	0.218
Liver cirrhosis, %	31.6	27.5	0.708	37.5	29.2	0.516
Esophageal varices, %	17.3	13.7	0.644	22.9	14.6	0.433
*Preoperative laboratory factors*						
HBsAg + , %	15.3	19.6	0.408	20.8	18.8	1.000
HCVAb + , %	51.0	49.0	0.864	52.1	52.1	1.000
Hemoglobin, g/dL, mean	13.5	13.6	0.857	13.5	13.5	0.997
Platelets, 10^4^/μL, mean	15.8	16.7	0.596	15.3	16.6	0.466
Albumin, g/dL, mean	4.1	4.0	0.379	4.1	4.1	0.974
Bilirubin, mg/dL, mean	0.66	0.71	0.307	0.65	0.72	0.206
AST, IU/L, mean	43.6	46.4	0.252	47.4	46.6	0.809
Prothrombin, %, mean	94.4	95.7	0.671	95.0	95.5	0.558
ICG-R_15_, %, mean	13.6	14.3	0.573	14.5	14.5	0.590
Child–Pugh, A, %	98.0	100	0.547	100	100	NS
*Surgical factors*						
Surgical time, minutes, mean	348.3	350.5	0.986	341.5	350.6	0.977
Pringle’s time, minutes, mean	78.3	89.8	0.202	78.1	88.9	0.416
Blood loss, mL, mean	325.7	464.9	0.033	340.1	473.1	0.033
Blood transfusion, mL, mean	25.7	60.4	0.398	25.7	60.4	0.979
*Tumor-related factors*						
Tumor number, single, %	82.7	82.4	1.000	81.3	83.3	1.000
Tumor size, cm, mean	3.3	3.2	0.599	3.3	3.0	0.600
Surgical margin, mm, mean	6.3	6.4	0.965	5.0	6.5	0.534
Complications, n (%)	40 (40.8)	17 (33.3)	0.478	21 (43.8)	17 (35.4)	0.532
Clavien–Dindo grade			1.000			1.000
Grade I–IIIa, %	93.9	94.1		93.8	93.8	
Grade IIIb–V, %	6.1	5.9		6.3	6.3	
Morbidity and mortality						
30-day mortality, %	1.0	0	1.000	0	0	NS
Surgical mortality, %	0	2.0	0.342	0	1.0	1.000
Hospital stay, days, mean	14.3	14.0	0.163	14.5	14.3	0.382

OS hepatectomy; hepatic segmentectomy so-called one-segment hepatectomy; *PS* performance status, *BMI* body mass index, *HBsAg* hepatitis B surface antigen, *HCVAb* hepatitis C virus antibody, *AST* Aspartate transaminase; *ICG-R15* indocyanine green retention rate at 15 min, *DCP* des-gamma-carboxyprothrombin, *NS* not significant

### Impact of expert surgeons on MOSeg hepatectomies

In the second period, expert surgeons participated in 94 MOSeg hepatectomies and did not participate in 35. Propensity score matching was used to evenly distribute 26 patients to the expert surgeon participation and non-participation groups (Table 3). No significant difference was found for surgical factors, complications, mortality, or overall survival between the two groups (Fig. 2d).

**Table 3:** Impact of expert surgeon participation on MOS hepatectomy before and after propensity score matching

Characteristic	Before matching			After matching		
	Participation (*n* = 94)	Non-participation (*n* = 35)	*P* value	Participation (*n* = 26)	Non-participation (*n* = 26)	*P* value
*Profiles*						
Age, years, mean	66.2	68.7	0.393	68.3	68.6	0.653
Male sex, %	83.0	74.3	0.318	76.9	88.5	0.465
PS 0, %	98.9	97.1	0.471	100	96.2	1.000
BMI, mean	23.2	25.2	0.014	23.3	24.1	0.234
*Comorbidity*						
Alcohol habitually, %	27.7	25.7	1.000	30.8	34.6	1.000
Smoking, %	60.6	62.9	0.842	65.4	73.1	0.764
Liver cirrhosis, %	5.3	11.4	0.253	0	11.5	0.235
Esophageal varices, %	4.3	2.9	1.000	0	3.8	1.000
*Preoperative laboratory factors*						
HBsAg + , %	24.5	8.6	0.051	19.2	11.5	0.703
HCVAb + , %	24.5	25.7	1.000	11.5	23.1	0.465
Hemoglobin, g/dL, mean	13.6	13.8	0.998	13.5	13.9	0.641
Platelets, 10^4^/μL, mean	22.5	21.5	0.287	21.4	22.2	0.654
Albumin, g/dL, mean	4.0	4.0	0.861	4.0	3.9	0.315
Bilirubin, mg/dL, mean	0.74	0.58	0.003	0.62	0.61	0.680
AST, IU/L, mean	46.9	40.7	0.170	42.8	42.9	0.687
Prothrombin, %, mean	93.8	96.7	0.066	94.3	96.4	0.185
ICG-R_15_, %, mean	12.2	9.5	0.937	11.7	11.3	0.167
Child–Pugh, A, %	96.8	100	0.562	100	100	NS
*Surgical factors*						
Surgical time, minutes, mean	453.4	418.3	0.137	436.1	424.0	0.891
Pringle’s time, minutes, mean	99.4	100.3	0.861	93.6	104.4	0.297
Blood loss, mL, mean	766.3	635.6	0.951	430.9	638.4	0.082
Blood transfusion, mL, mean	126.0	32.0	0.234	27.7	32.3	0.960
*Tumor-related factors*						
Tumor number, single, %	73.4	62.9	0.280	80.8	61.5	0.220
Tumor size, cm, mean	8.4	7.0	0.113	6.8	7.9	0.336
Surgical margin, mm, mean	3.1	4.3	0.157	4.8	4.3	0.697
Complications, n (%)	35 (37.2)	14 (40.0)	0.839	7 (26.9)	12 (46.2)	0.249
Clavien–Dindo grade			1.000			1.000
Grade I–IIIa, %	95.7	97.1		96.2	96.2	
Grade IIIb–V, %	4.3	2.9		3.8	3.8	
*Morbidity and mortality*						
30-day mortality, %	0	0	NS	0	0	NS
Surgical mortality, %	0	1.1	1.000	0	0	NS
Hospital stay, days, mean	15.9	13.9	0.562	16.4	14.0	0.706

MOS hepatectomy; hepatectomy of more than one segment including hepatic trisectionectomy, hemihepatectomy, central bisectionectomy of the liver, hepatic sectionectomy (except for lateral sectionectomy); *PS* performance status, *BMI* body mass index, *HBsAg* hepatitis B surface antigen, *HCVAb* hepatitis C virus antibody, *AST* Aspartate transaminase, *ICG-R15* indocyanine green retention rate at 15 min, *DCP* des-gamma-carboxyprothrombin, *NS* not significant

## Discussion

This study focused on high-level liver surgery for HCC to assess the significance of the JBCSES. In terms of surgical outcomes in the periods before and after the establishment of the system, surgical times for hepatectomies in the second period were longer than those in the first period, but they involved significantly lower intraoperative blood loss and transfusion volumes. The incidence of complications and frequency of severe complications decreased. OSeg hepatectomies that involved the participation of expert surgeons had significantly lower blood loss than those that did not. These findings suggest that the introduction of the JBCSES played an important role in promoting safer surgeries and that the participation of expert surgeons contributes to lesser blood loss in OSeg hepatectomies.

This study’s results raised four important questions. The first relates to the decreasing trend in complications and the improvement in patient survival rates in the second period. The most important reason for this is the steep learning curve in performing hepatectomies [28, 29]. In the first period, surgeons lacked sufficient experience with the transection method, and only 12 high-level liver surgeries were performed annually, on average, in our hospital. In the second period, parenchymal transection using the Pean forceps fracture method and the technique facilitating hepatectomies without bleeding became well-established. In the second period, the operating times were longer because of careful dissection. Anesthesia techniques and advances in perioperative management have also contributed. The number of high-level liver surgeries also doubled to 23 annually, on average, and our hospital became a high-volume hospital in Japan, which also explains the fewer complications. One “Japanese board-certified training institution A” criterion is that ≥50 high-level hepato-biliary-pancreatic surgeries, including hepatectomy in HCC, liver metastasis, and bile duct carcinoma, and pancreatectomy in liver and pancreatic cancers, must be performed annually [1]. Recently, the incidence of hepatitis C and HCC has plateaued in Japan and Europe and is decreasing in some regions [30, 31]. The number of hepatectomies in HCC is decreasing in Japan, and opportunities to learn about hepatectomy for liver conditions, such as cirrhosis, will likely decrease. Therefore, developing ways to teach surgeons safe hepatectomy methods for patients with poor liver function will be a challenge in the future.

### Japanese HBP expert surgeon

The JBCSES was established to improve surgical safety and patient prognosis, and the advent of this system has undoubtedly improved the safety awareness of all surgeons performing liver-related surgeries, which could explain the reduction in complications and bleeding.

The second question relates to the equal incidence of complications in OSeg and MOSeg hepatectomies in the second period. Among the 11,000 patients in the NCD, more complications occurred in MOSeg than in OSeg hepatectomies because the former involves a greater extent of resection [32], whereas OSeg hepatectomies require more advanced and delicate techniques [9, 14]. We adhered strictly to the resection criteria and consciously selected the less-extensive resection method when the ICG-R_15_ value was on the border of OSeg and MOSeg hepatectomies [33]. Therefore, it is possible that selecting the less extensive hepatectomy for safety helped minimize postoperative complications.

The third question relates to the assessment of expert surgeons. A study comparing board-certified and non-board-certified training institutions reported that the former were superior in terms of decreasing blood loss, transfusion volumes, complications, and 30-day mortality [2]. In high-volume centers, such as ours, surgeons may improve their techniques uniformly [34], which may explain the uniformity in the incidence of complications or cumulative survival, regardless of expert surgeon participation in OSeg and MOSeg hepatectomies. After propensity score matching, the significantly lower blood loss volume in OSeg hepatectomies with expert surgeon participation represents a beneficial outcome of the JBCSES. Less blood loss stabilizes postoperative management and likely reduces the incidence of complications; thus, blood loss is an effective index for the technical assessment of expert surgeons in all institutions, including high-volume centers. It is unknown, however, why there was a significant difference in bleeding volume only in OSeg hepatectomies, not in both types of hepatectomies. For MOSeg hepatectomies, no technical difference could explain a practical difference in the bleeding volumes between the participation and non-participation groups. For MOSeg hepatectomies, the surface after liver resection is flat, making it a relatively simple hepatic transection operation. However, for OSeg hepatectomies, the surface after liver resection is three-dimensional, and hepatic transection is difficult, requiring the exposure of multiple hepatic veins, which may explain the different volumes of bleeding during surgery. Although another study showed that the participation of three or more expert surgeons contributed to a low 90-day postoperative mortality rate in patients who had undergone all types of hepatectomies [35], that research did not investigate the relevance of expert surgeons participating in high-level hepatectomy.

The fourth question relates to the study’s limitations. The results were derived from data from one high-volume hospital. To evaluate the generalizability of the JBCSES’s effects, more investigations involving board-certified institutions throughout Japan are required. Although it was possible to evaluate postoperative complications between OSeg and MOSeg hepatectomies using NCD data, information about expert surgeon participation was absent; we propose the addition of such information as a new variable in the database. Ideally, randomized controlled trials investigating the effectiveness of high-level liver surgery and expert surgeons should be conducted. Otsubo et al. reported that the annual mortality rates for all high-level HBP surgeries significantly decreased in 2011–2015 [1]. The 30-day mortality rates in 2012, 2013, 2014, and 2015 were 0.9%, 0.7%, 0.6%, and 0.6%, respectively. However, the number of surgeries for HCC per board-certified institution from 2012 to 2015 was 12–15 cases annually in that study, which was considerably fewer than our 23 cases. To further improve the expertise level of hepatectomies, specific HCC or high-risk patients should be treated at high-volume hospitals. Furthermore, neither laparoscopic surgery nor the use of new surgical devices was included in our research. Recently, these have been adopted for hepatectomies in many institutions. We believe that further examination of the role of expert surgeons in OSeg and MOSeg hepatectomies, including laparoscopic surgery and surgeries utilizing new surgical devices, is necessary. This study has a small number of cases; hence, further investigations should include more cases.

## Conclusions

The requirements for the Japanese board certification system for expert surgeons to perform high-level hepatectomy for HCC were appropriate and might decrease the complications of high-level hepatectomy in HCC and improve long-term survival. In particular, the participation of expert surgeons in OSeg hepatectomy significantly decreased intraoperative blood loss.
